# *TERT* promoter mutations and Ki-67 labeling index as a prognostic marker of papillary thyroid carcinomas: combination of two independent factors

**DOI:** 10.1038/srep41752

**Published:** 2017-02-02

**Authors:** Michiko Matsuse, Tomonori Yabuta, Vladimir Saenko, Mitsuyoshi Hirokawa, Eijun Nishihara, Keiji Suzuki, Shunichi Yamashita, Akira Miyauchi, Norisato Mitsutake

**Affiliations:** 1 Department of Radiation Medical Sciences, Atomic Bomb Disease Institute, Nagasaki University, Nagasaki, Japan.; 2 Department of Surgery, Kuma Hospital, Kobe, Japan; 3 Department of Radiation Molecular Epidemiology, Atomic Bomb Disease Institute, Nagasaki University, Nagasaki Japan; 4 Department of Diagnostic Pathology, Kuma Hospital, Kobe, Japan.; 5 Department of Internal Medicine, Kuma Hospital, Kobe, Japan

## Abstract

Although most papillary thyroid carcinomas (PTCs) have a good prognosis, a small but certain fraction shows aggressive behavior. Therefore, a novel and well-performing molecular marker is needed. In the present study, we assessed the impact of the combination of the *TERT* promoter/*BRAF* mutations and Ki-67 labeling index (LI) as a prognostic marker in PTC patients. Of 400 PTC samples, 354 were successfully genotyped for both *TERT* promoter/*BRAF* and analyzed for Ki-67 LI. Based on the combination of the mutational status and Ki-67 LI, the cases were categorized into three groups: high-, middle-, and low-risk. The recurrence rates of low-, middle-, and high-risk group were 1.9% (6 of 323), 18.2% (4 of 22), and 44.4% (4 of 9), respectively. The Kaplan-Meier curve and log-rank analyses demonstrated that there were statistical differences between any two groups. The hazard ratios for recurrence remained significant after adjustment for age, sex, tumor size, and extrathyroidal extension (low vs. middle: 8.80, 95% CI: 2.35–32.92, *p* = 0.001; middle vs. high: 6.255, 95% CI: 1.13–34.51, *p* = 0.035). In conclusion, the combination of the *TERT* promoter/*BRAF*^*V600E*^ mutations and Ki-67 LI performed excellent in predicting PTC recurrence and may be clinically useful.

Papillary thyroid carcinoma (PTC) is the most common malignant tumor in thyroid. Although most PTCs have a good prognosis, a small but certain fraction shows aggressive behavior, and recurrence occurs in 5–20% of the cases^1^. To discriminate such high-risk cases from others, a number of studies have been conducted to find prognostic molecular markers.

The *BRAF*^*V600E*^ mutation is the most prevalent genetic alteration in PTCs, ranging 29–83%, 44% on average^2^. Many studies have demonstrated that the presence of the *BRAF*^*V600E*^ mutation is associated with aggressive characteristics of the disease, such as extrathyroidal invasion, lymph node metastasis, and a poor prognosis^3^,^4^,^5^,^6^. On the other hand, there are also some studies suggesting no relationship between *BRAF*^*V600E*^ and tumor aggressiveness^7^,^8^,^9^. The mutation rates reported from East Asian countries such as Japan and South Korea are generally high (~80%), but PTCs in these countries do not seem to have a worse prognosis. The significance of this mutation as a prognostic marker is still controversial and may be dependent on a population.

Recently, two recurrent somatic mutations, chr5:1,295,228 C>T (C228T) and chr5:1,295,250 C>T (C250T), in the promoter region of the telomerase reverse transcriptase (*TERT*) gene have been found in various types of cancers including thyroid cancer^10^,^11^,^12^, which represent nucleotide changes of –124 C>T and –146 C>T from the ATG translation start site, respectively. These mutations generate binding sites for E-twenty-six (ETS) transcription factors and have been shown to increase the transcriptional activity of the *TERT* promoter by two- to four-fold^13^,^14^. Telomerase is a ribonucleoprotein enzyme that synthesizes telomeric DNA. Telomerase is not active in most adult tissues. However, it is reactivated in many cancers through the transcriptional regulation of the *TERT* gene which encodes a key component of the telomerase complex^15^. The TERT protein is thought to play an important role in human tumorigenesis. Its canonical function is to maintain telomere length, preventing replicative senescence. Recent studies have also suggested that TERT has novel molecular functions including transcriptional regulation of NF-κB and Wnt/β-catenin target genes involved in cell proliferation, resistance to apoptosis, invasion, and metastasis^16^. In thyroid tumors, the *TERT* promoter mutations have been shown to be associated with aggressive features in the presence of the *BRAF* or *RAS* mutation^17^,^18^,^19^,^20^,^21^,^22^,^23^,^24^. Recently, Xing *et al*. have shown that the coexistence of *BRAF*^*V600E*^ and *TERT*^*C228T*^ is correlated with high-risk clinicopathological characteristics and a worse prognosis^24^.

Ki-67 is a nuclear protein that is associated with cellular proliferation. Although little is known about what Ki-67 actually does, it is present during all active phases of the cell cycle but absent in resting (G0) cells. The expression of Ki-67 protein is evaluated as a labeling index (LI) on tissue specimens. A high Ki-67 LI was associated with a poor prognosis in patients with breast cancer and prostate cancer^25^. In PTCs, it has been reported that the Ki-67 LI is an independent prognostic factor for disease-free and cause-specific survival^26^,^27^.

In this study, we set out to clarify the prognostic value of *BRAF*^*V600E*^ and the *TERT* promoter mutations in Japanese PTCs, in which the prevalence of the *BRAF*^*V600E*^ mutation is very high. Moreover, we also tried to combine the mutational status with the Ki-67 LI to achieve better prediction.

## Results

### Mutational status of the BRAF gene and the TERT promoter

DNA samples were first screened for the *BRAF* mutation. Of 398 samples, 383 (96.2%) were successfully genotyped, of which 313 (81.7%) and 4 (1.04%) were found to carry the *BRAF*^*V600E*^ and the *BRAF*^*V600delinsYM*^ mutations^28^, respectively, and 66 (17.2%) were negative for the mutations. We next analyzed the promoter region of *TERT*. Of 398 samples, 363 samples were available for the test because of limited amount of DNA. Of 363 samples, 357 (98.3%) were successfully genotyped. The *TERT* promoter mutations were found in 36 (10.1%) samples, among which *TERT* C228T was more frequent (33 of 36) than C250T (3 of 36), and the two mutations were mutually exclusive. Of these 357 samples, 356 were successfully genotyped for the *BRAF* status. In our series, all *TERT* promoter mutation-positive PTC samples harbored the *BRAF*^*V600E*^ mutation. There was a significantly higher co-occurrence between the *TERT* promoter mutation and the *BRAF*^*V600E*^ mutation (*p* < 0.001; Fisher’s exact test, two-sided). Genotyping results were classified into three groups: *BRAF*^*V600E*^ alone, 258 patients (72.5%); *TERT* promoter mutation-positive group (all of these harbored the *BRAF*^*V600E*^ mutation), 36 patients (10.1%); and mutation-negative group: 62 patients (17.4%).

### Relationship between the mutational status and clinicopathological features

**Figure 1:**
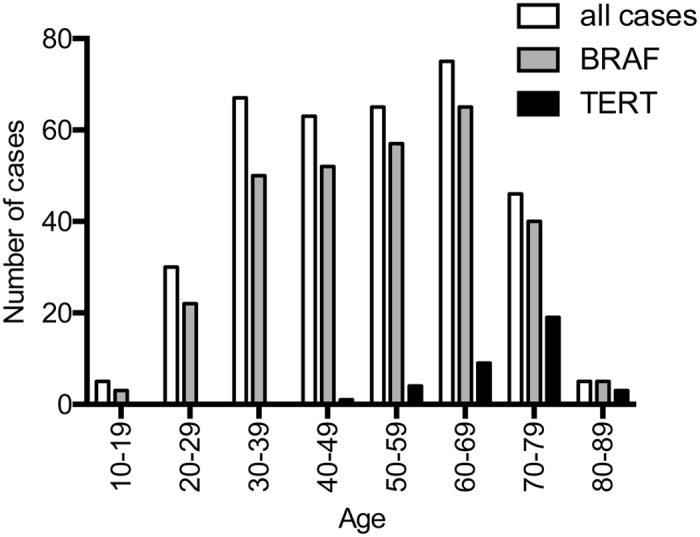
Age distribution of the *BRAF* and *TERT* promoter mutations.

We analyzed the association of *BRAF*^*V600E*^ alone and coexistence of *BRAF*^*V600E*^ and the *TERT* promoter mutation with clinicopathological characteristics. Unfortunately, in this series it was not possible to address the effect of the *TERT* promoter mutation alone due to absence of such cases. As shown in Table 1, the mean age was significantly older in the *TERT* promoter mutation-positive group (70.1 ± 8.3 years old) than in the *BRAF*^*V600E*^ alone (49.7 ± 14.6 years old, *p* < 0.001) or mutation-negative group (45.8 ± 16.6 years old, *p* < 0.001). Interestingly, the *TERT* promoter mutation was not found at all in patients less than 45 years old, and its prevalence was substantially increased with age afterwards (Fig. 1). The tumor size was significantly greater in the *TERT* promoter mutation-positive group (32.1 ± 15.7 mm) than in the *BRAF*^*V600E*^ alone (19.8 ± 11.4 mm, *p* < 0.001) or mutation-negative group (22.6 ± 15.3 y, *p* = 0.001) (Table 1). Distant metastasis, advanced stage, and extrathyroidal invasion were also more common in the *TERT* promoter mutation-positive group while no difference was found for nodal disease frequency among the groups (please see Table 1 for details). These data demonstrate that the *TERT* promoter mutations were associated with the aggressive clinicopathological characteristics of PTC.

### Relationship between Ki-67 LI and clinicopathological features

Of 400 samples, 395 (98.7%) were successfully analyzed for the Ki-67 LI. The patients were categorized into three groups: LI <5%, 304 patients (77.0%); LI 5–10%, 68 patients (17.2%); and LI> 10%, 23 patients (5.8%) (Table 2). Analysis of clinicopathological relevance of Ki-67 LI did not detect significant associations after corrections for multiple comparisons (Table 2). The only statistically significant trend was found for distant metastasis (*p* = 0.018, the Cochran-Armitage test).

### Relationship between the mutational status and recurrence

**Figure 2:**
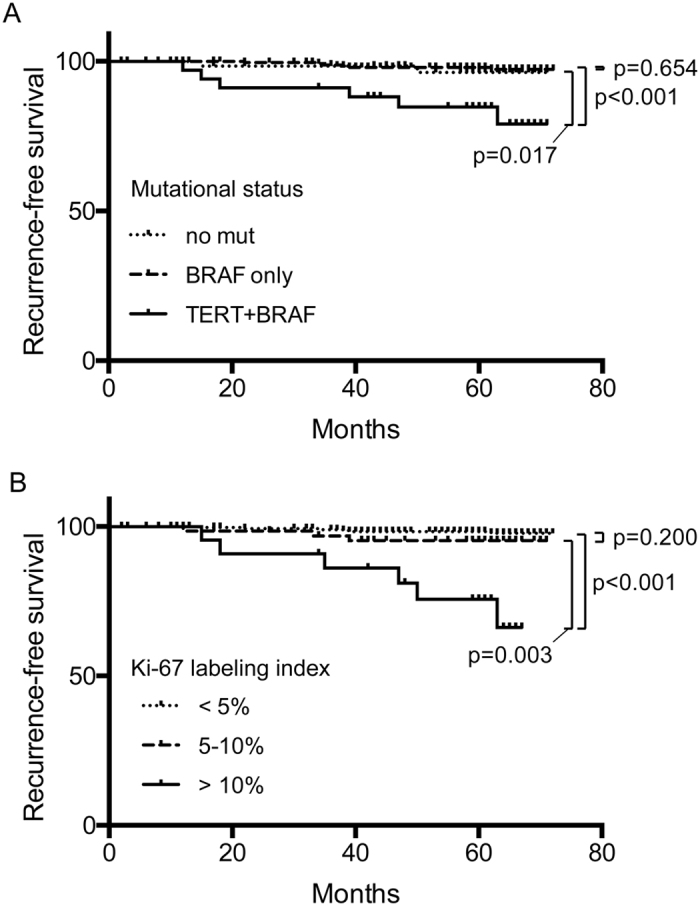
Kaplan-Meier curves of recurrence-free survival. The vertical tick-marks correspond to censored data. *p*-values of a log-rank test are shown. (**A**) by mutational status. (**B**) by Ki-67 labeling index.

We next assessed the impact of the *TERT* promoter mutations on disease recurrence. Compared to patients with *BRAF*^*V600E*^ (6 of 258, 2.3%) alone and without mutations (2 of 62, 3.2%), those with both *BRAF*^*V600E*^ and *TERT* promoter mutations had a higher recurrence rate (6 of 36, 16.7%). We then performed Kaplan-Meier analysis and log-rank test of recurrence-free survival of the patients. The presence of both *BRAF*^*V600E*^ and *TERT* promoter mutations was significantly associated with recurrence (Fig. 2A, *p* = 0.017 vs. no mutation, *p* < 0.001 vs. *BRAF*^*V600E*^ only). In contrast, we did not see any difference at all between *BRAF*^*V600E*^ alone and no mutation groups (Fig. 2A). The hazard ratio (HR) for tumor recurrence in patients with *BRAF*^*V600E*^ and *TERT* promoter mutations was 6.74 (95% CI: 2.17–20.91, *p* = 0.001) (Table 3, upper), which remained significant after adjustment for patient age, sex, and lymph node metastasis (HR: 4.17, 95% CI: 1.06–17.7, *p* = 0.048) (Table 3, upper). However, when tumor size or extrathyroidal extension was included in the adjustment, statistical significance was lost (Table 3, upper). Again, we did not find any significant difference between mutation-negative group and *BRAF*^*V600E*^-positive group (Table 3, upper). In addition, ROC curve analysis demonstrated that, based on the mutation type definition (i.e., no mutation, *BRAF*^*V600E*^ only, or *TERT* promoter/*BRAF*^*V600E*^), the area under curve (AUC) was 0.672 (SD = 0.096, accuracy 28.6%).

### Relationship between the Ki-67 LI and recurrence

The recurrence rates of the LI <5%, the LI 5–10%, and the LI> 10% groups were 2.0% (6 of 304), 4.4% (3 of 68), and 26.1% (6 of 23), respectively. Kaplan-Meier and log-rank analyses demonstrated that the LI> 10% group showed significantly worse recurrence-free survival (p < 0.001 vs LI <5%, p = 0.003 vs LI 5–10%) (Fig. 2B). However, there was no statistical difference between LI <5% and LI 5–10%. The HR for recurrence in patients with LI> 10% was 9.87 (95% CI: 3.42–28.5, *p* < 0.001), which remained significant after adjustment for age, sex, lymph node metastasis, tumor size, and extrathyroidal extension (HR: 5.521, 95% CI: 1.72–17.74, *p* = 0.004) (Table 3, middle). The HRs of those with LI 5–10% were 2.44 (unadjusted; 95% CI: 0.61–9.78, *p* = 0.207), 2.62 (adjusted for age, sex, and lymph node metastasis; 95% CI: 0.65–10.56, *p* = 0.176), and 2.25 (adjusted for age, sex, lymph node metastasis, tumor size, and extrathyroidal extension; 95% CI: 0.55–9.22, *p* = 0.261), all of which were not statistically significant though. ROC curve analysis of recurrence based on the three categories of Ki-67 LI returned AUC of 0.727 (SD = 0.120, accuracy 77.7%).

### Combination of the TERT promoter mutations and the Ki-67 LI

**Figure 3:**
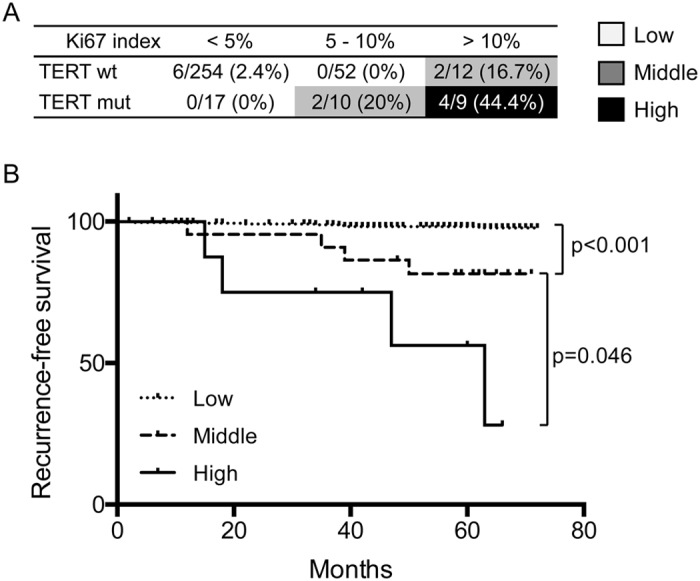
(**A**) Classification of risk group using the mutational status and the Ki-67 labeling index and recurrence rates. (**B**) Kaplan-Meier curves of recurrence-free survival by risk groups. The vertical tick-marks correspond to censored data. *p*-values of a log-rank test are shown.

As described above, the associations of the *TERT* promoter mutations with the clinicopathological parameters were stronger as compared to those of the Ki-67 LI. Therefore, we combined both mutational status and LI to re-categorize the patients into three following groups: low-risk group, *TERT* mutation negative/LI ≤10% and *TERT* mutation positive/LI <5%; middle-risk group, *TERT* mutation negative/LI> 10% and *TERT* mutation positive/LI 5–10%; high-risk group, *TERT* mutation positive/LI> 10% (Fig. 3A). The recurrence rates of low-, middle-, and high-risk group were 1.9% (6 of 323), 18.2% (4 of 22), and 44.4% (4 of 9), respectively (Fig. 3A). As shown in Fig 3B, the Kaplan-Meier curve of the low-risk group was excellent; that of the high-risk group was the worst; and that of the middle-risk group was in between. There were statistical differences between any two groups (Fig. 3B). The HRs for recurrence were statistically significant (low vs. middle: 10.15, 95% CI: 2.86–36.01, *p* = 3.36E-04; middle vs. high: 11.76, 95% CI: 3.64–38.05, *p* = 3.87E-05), which remained significant after adjustment for age, sex, tumor size, and extrathyroidal extension (low vs. middle: 8.80, 95% CI: 2.35–32.92, *p* = 0.001; middle vs. high: 6.255, 95% CI: 1.13–34.51, *p* = 0.035) (Table 3, lower). ROC curve analysis based on the three risk categories determined AUC of 0.889 (SD = 0.072, accuracy 91.8%).

## Discussion

The prevalence of the *BRAF*^*V600E*^ mutation in PTCs in East Asian countries including Japan is high. The prevalence of the *BRAF*^*V600E*^ mutation was reported to be dependent on the amount of iodine intake^29^; however, very recently it has been demonstrated that there was no difference in the *BRAF*^*V600E*^ prevalence between Japan, an iodine-rich country, and Vietnam, an iodine-deficient country^30^. Interestingly, in the meta-analysis by Kim *et al*.^31^, when they divided analyzed studies into two groups according to the prevalence of the *BRAF*^*V600E*^ mutation (≥50% and <50%), the pooled effect sizes of extrathyroidal invasion and lymph node metastasis were about 30% smaller in the *BRAF*^*V600E*^ ≥50% group than in the <50% group. The impact of the *BRAF*^*V600E*^ mutation on aggressive clinicopathological features might depend on the prevalence of the mutation. In the present study, the association of the *BRAF*^*V600E*^ mutation alone with the aggressive clinicopathological features and recurrence was negative. The reason for the difference in the impact of the *BRAF*^*V600E*^ mutation remains to be further addressed.

The rate of the *TERT* promoter mutations (C288T and C250T) in the current series was 10.1%, which is consistent with most of the previous studies 32,33. There seems to be no large difference between populations except for two studies from China reporting 4.1 and 4.4%^19^,^23^, perhaps the smallest two among published data. The presence of the *TERT* promoter mutation only was also reported to be associated with PTC aggressiveness^24^; however, in the present study we were not able to analyze this because we did not have even one case with the *TERT* promoter mutation but without *BRAF*^*V600E*^, which is a limitation of our work. To assess the impact of the *TERT* promoter mutation alone in the *BRAF*^*V600E*^ prevalent region such as Japan, it would be necessary to collect a very large number of samples. This is a future issue but cases with *TERT* promoter mutation alone probably show better prognosis than those with the *TERT* promoter plus *BRAF*^*V600E*^ mutations. A number of studies have demonstrated that the co-existence of the *BRAF*^*V600E*^ and *TERT* promoter mutations are associated with aggressive clinicopathological features and disease recurrence^17^,^18^,^19^,^20^,^21^,^22^,^23^,^24^. As far as we know, there is no report against the above finding so far. The result of the present study also supports this. We also observed that the presence of the *TERT* promoter mutations showed very strong age dependency, and it is noteworthy that the mutations were not detected in any of the patients less than 45 years old. Nevertheless, the HR for recurrence after adjustment for age was still statistically significant.

The *TERT* promoter mutations were linked to many aggressive clinicopathological features such as large tumor size, distant metastasis, advanced stage, and extrathyroidal extension. Therefore, it was not too surprising that the statistical significance of the increased HR for recurrence was lost after adjustment for tumor size or extrathyroidal extension in addition to age and sex. However, as their effect sizes were still relatively high (3.016 [adjusted for age, sex, and size], 4.068[adjusted for age, sex, Ex]), the number of samples was perhaps not enough to reach statistical significance. Our ROC curve analysis demonstrated that mutation type would not be a good test for predicting recurrence in the series under analysis (AUC = 0.672). This is a first study reporting a clinical significance of the *BRAF*^*V600E*^ and *TERT* promoter mutations in the Japanese PTC cases, which show higher *BRAF*^*V600E*^ prevalence (81.7% in this study). This is consistent with the recent report from South Korea where the prevalence of the *BRAF*^*V600E*^ mutation is also high^20^.

It has been demonstrated that the Ki-67 LI is associated with a prognosis of PTC^26^,^27^. The current study also confirmed these observations. Indeed, the HR for recurrence of the Ki-67 LI> 10% was significant even after adjustment for a number of parameters, and effect size was greater than that for the *TERT* promoter/*BRAF*^*V600E*^ mutations. Also, ROC curve analysis indicated that Ki-67 LI performed better than mutation type as a test for predicting recurrence (AUC = 0.727).

Interestingly, the associations of the Ki-67 LI with the clinicopathological characteristics were much weaker than those of the *TERT* promoter/*BRAF*^*V600E*^ mutations, suggesting that Ki-67 LI may be a prognostic marker that is associated with higher-risk tumors independently of their mutational status. Indeed, the Ki-67 LI was reported to have an inverse correlation with the thyroglobulin-doubling time, suggesting that it reflects growth velocity of tumor^27^. On the other hand, the *TERT* promoter/*BRAF*^*V600E*^ mutations are strongly linked to the clinicopathological status. They may be independent factors. We, therefore, combined the mutational status and the Ki-67 LI and divided the cases into three risk groups and found 6–8-fold adjusted differences in effect sizes between the groups. Concordantly, the ROC curve analysis based on the risk groups demonstrated further improvement of the test performance (AUC = 0.889). It should be noted that, despite the presence of the *TERT* promoter/*BRAF*^*V600E*^ mutations, there was no recurrence in the patients with Ki-67 LI <5% (low-risk group). In contrast, 16.7% of the PTCs recurred if Ki-67 LI was >10% even though the tumors did not carry the *TERT* promoter/*BRAF*^*V600E*^ mutations (middle-risk group). Approximately half of the high-risk patients had disease recurrence, although the number of such patients was rather small. Thus, neither mutational status nor Ki-67 LI was the optimal predictor for recurrence. Our statistical calculations showed that the combination of these risk factors performs better, yet ample room for improvement still remains.

In conclusion, the combination of the *TERT* promoter/*BRAF*^*V600E*^ mutations and Ki-67 LI is a promising marker to predict recurrence of PTC. Low-, middle-, or high-risk group showed clear differences in any of the comparisons. The limitations of this method are: 1) The Ki-67 LI can only be obtained using surgical specimens, 2) It may be difficult to compare the Ki-67 LI from different institutions. Therefore, it is desired to find an objective marker which is strongly correlated with Ki-67 LI that can be measured preoperatively.

## Methods

### PTC samples

A total of 400 adult sporadic PTC samples were collected at Kuma Hospital (Kobe, Japan). All patients received surgical treatment in 2009, and the therapeutic strategy was not changed during whole treatment course. Histological diagnosis was performed by a thyroid pathologist (MH). All patients had no history of radiation exposure. Patients’ age at operation ranged 13–87 years old (mean 51 ± 16, median 52 years old, 15.2% male); follow-up period was 2–72 months (mean 58 ± 14, median 63 months). Disease recurrence was defined as surgically removed and pathologically verified local tumor focus or regional metastasis, or distant metastasis detected by ultrasound or radioisotope imaging not earlier than 12 months after initial treatment. According to this criterion, we excluded one case that had a recurrence within three months. The study protocol was approved by the ethics committees of Nagasaki University and Kuma Hospital. All procedures were performed in accordance with the relevant guidelines and regulations.

### DNA extraction and mutation screening

DNA was extracted from formalin-fixed paraffin-embedded (FFPE) PTC tissues using a QIAamp DNA mini kit (QIAGEN) according to the manufacturer’s protocol. DNAs of sufficient quality and quantity for sequencing were obtained from 398 of 400 PTC specimens. *BRAF* (around V600) and *TERT* promoter mutations were analyzed by direct DNA sequencing. Primer sequences used for both PCR amplification and sequencing are: BRAF-FW, 5′-ACATACTTATTGACTCTAAGAGGAAAGATGAA-3′; BRAF-RV, 5′-GATTTTTGTGAATACTGGGAACTATGA-3′; TERT-FW, 5′-CAGCGCTGCCTGAAACTC-3′; and TERT-RV, 5′-GTCCTGCCCCTTCACCTT-3′^22^. First, PCR amplification was done using KOD FX (TOYOBO). PCR products were then treated with ExoSAP-IT PCR clean-up reagent (GE Healthcare), and sequencing was performed with a Big Dye Terminator sequencing kit version 3.1 (Applied Biosystems) on an ABI3730 automated sequencer (Applied Biosystems). We prepared one negative control (without tissue section) per every 23 samples during DNA extraction to ensure contamination-free amplifications.

### Ki-67 immunohistochemistry and the LI

Immunostaining was performed using 4-μm-thick FFPE sections of the same PTC cases. Anti-Ki-67 antibody (clone MIB1, Dako) was used as a primary antibody. The staining was carried out using the Dako Cytomation Autostainer Universal System (Dako) and the Envision kit (Dako) according to the manufacturer’s instruction. A single pathologist (MH) evaluated the specimens without clinical information of the patient. To obtain the Ki-67 LI, at least 500 carcinoma cells in hot areas were analyzed under × 400 magnification. Staining results were classified into three groups:<5%, 5%–10%, and >10% of positive cells.

### Statistical analysis

Statistical analysis was performed using SPSS software version 21.0.0.0 (IBM), GraphPad Prism version 6.0 (GraphPad Software), and SAS University Edition software (SAS Institute Inc). For multiple comparisons, the nonparametric one-way ANOVA with Dunnett *post hoc* test or the FREQ and COMPPROP procedures (SAS) were used for continuous variables or group analyses, respectively. For univariate disease-free survival, the log-rank and the Kaplan-Meier estimates were calculated, and the Cox proportional hazard model was applied in multivariate analyses. The Receiver Operating Characteristic (ROC) curve analysis was performed under parametric distribution assumption (Eng J. ROC analysis: web-based calculator for ROC curves. Baltimore: Johns Hopkins University. Available at: http://www.jrocfit.org/, accessed on July 12, 2016). The *p* value less than 0.05 was regarded as indicating statistical significance.

## Additional Information

**How to cite this article**: Matsuse, M. *et al. TERT* promoter mutations and Ki-67 labeling index as a prognostic marker of papillary thyroid carcinomas: combination of two independent factors. *Sci. Rep.* **7**, 41752; doi: 10.1038/srep41752 (2017).

**Publisher's note:** Springer Nature remains neutral with regard to jurisdictional claims in published maps and institutional affiliations.

## Figures and Tables

**Table 1:** Association between mutational status and clinicopathological findings.

Genotype		No BRAF, no TERT (1)	BRAF mut (2)	BRAF/TERT mut (3)	p-value (1 vs 2)	p-value (2 vs 3)	p-value (1 vs 3)
Number of cases		62 (17.4%)	258 (72.5%)	36 (10.1%)			
Age (mean ± s.d.)		45.8 ± 16.6	49.7 ± 14.6	70.1 ± 8.3	0.259^a^	<**0.001**^a^	<**0.001**^a^
Sex (F/M, % male)		57/5 (8.1%)	218/40 (15.5%)	28/8 (22.2%)	ns^b^	ns^b^	ns^b^
Tumor size (mean ± s.d.)		22.6 ± 15.3	19.8 ± 11.4	32.1 ± 15.7	0.445^a^	**<0.001**^a^	**0.014**^a^
LN metastasis		34/61 (55.7%)	167/258 (64.7%)	20/36 (55.6%)	ns^b^	ns^b^	ns^b^
Distant metastasis^d^		0	1 (0.4%)	5 (13.9%)	n/p	<**0.05**^b^	n/p
Stage^c^					<**0.05**^b^	<**0.05**^b^	<**0.05**^b^
	I	37	114	2			
	II	3	5	0			
	III	16	99	16			
	IV	6	40	18			
Ex		41 (66.1%)	189 (73.3%)	33 (91.7%)	ns^b^	<**0.05**^b^	<**0.05**^b^

ns: not significant, p ≥ 0.05.

n/p: not performed.

^a^Non-parametric ANOVA with Dunnett *post hoc* test.

^b^Multiple comparison test for proportions (using the COMPROP procedure in SAS http://www2.sas.com/proceedings/sugi31/204-31.pdf).

^c^Calculations for Stage (I + II) vs. (III + IV).

^d^All metastatic sites were the lung.

**Table 2:** Association between Ki67 labeling index and clinicopathological findings.

Ki67 labeling index		<5% (1)	5–10% (2)	>10% (3)	p-value (1 vs 2)	p-value (2 vs 3)	p-value (1 vs 3)
Number of cases		304 (77.0%)	68 (17.2%)	23 (5.8%)			
Age (mean ± s.d.)		51.2 ± 15.2	48.7 ± 17.4	52.5 ± 19.5	0.622^a^	0.793^a^	0.985^a^
Sex (F/M, % male)		255/47 (15.5%)	61/7 (10.3%)	18/5 (21.7%)	ns^b^	ns^b^	ns^b^
Tumor size (mean ± s.d.)		20.8 ± 12.7	22.9 ± 13.1	29.5 ± 20.1	0.548^a^	0.380^a^	0.144^a^
LN metastasis		190/303 (62.7%)	43/68 (63.2%)	16/23 (69.6%)	ns^b^	ns^b^	ns^b^
Distant metastasis^d^		3 (1.0%)	1 (1.5%)	2 (8.7%)	ns^b^	ns^b^	ns^b^
Stage^c^					ns^b^	ns^b^	ns^b^
	I	128	35	9			
	II	8	0	0			
	III	115	23	5			
	IV	53	10	9			
Ex		212 (69.7%)	56 (82.4%)	17 (73.9%)	ns^b^	ns^b^	ns^b^

ns: not significant, p ≥ 0.05.

^a^Non-parametric ANOVA with Dunnett *post hoc* test.

^b^Multiple comparison test for proportions (using the COMPROP procedure in SAS http://www2.sas.com/proceedings/sugi31/204-31.pdf).

^c^Calculations for Stage (I + II) vs. (III + IV).

^d^All metastatic sites were the lung.

**Table 3:** Hazard ratios of disease recurrence.

Genotype	HR	95% CI	p-value	HR	95% CI	p-value	HR	95% CI	p-value	HR	95% CI	p-value
no BRAF, no TERT	1.000			1.000			1.000			1.000		
BRAF mut only	0.696	0.141–3.450	0.658	0.578	0.114–2.930	0.508	0.676	0.135–3.398	0.635	0.626	0.125–3.139	0.569
BRAF/TERT mut	6.740	2.173–20.905	**0.001**	4.172	1.011–17.218	**0.048**	3.016	0.659–13.797	0.155	4.068	0.966–17.129	0.056
Adjustment				age, sex, N			age, sex, size			age, sex, Ex		
Ki67 labeling index	HR	95% CI	p-value	HR	95% CI	p-value	HR	95% CI	p-value			
**<**5%	1.000			1.000			1.000					
5–10%	2.444	0.611–9.779	0.207	2.620	0.650–10.563	0.176	2.247	0.547–9.222	0.261			
>10%	9.871	3.422–28.476	**<0.001**	8.131	2.718–24.323	**<0.001**	5.521	1.719–17.737	**0.004**			
Adjustment				age, sex, N			age, sex, N, size, Ex					
Risk group	HR	95% CI	p-value	HR	95% CI	p-value						
Low	1.000			1.000								
Low vs. Middle	10.147	2.859–36.012	**3.36E-04**	8.795	2.349–32.924	**0.001**						
Middle vs. High	11.762	3.636–38.048	**3.87E-05**	6.255	1.134–34.505	**0.035**						
Adjustment				age, sex, size, Ex								
